# Group-wise ANOVA simultaneous component analysis for designed *omics* experiments

**DOI:** 10.1007/s11306-018-1369-1

**Published:** 2018-05-21

**Authors:** Edoardo Saccenti, Age K. Smilde, José Camacho

**Affiliations:** 1 Wageningen University & Research, Wageningen, the Netherlands; 2 University of Amsterdam, Amsterdam, the Netherlands; 3 University of Granada, Granada, Spain

**Keywords:** Analysis of variance, Designed experiments, Principal component analysis, Sparsity

## Abstract

**Introduction:**

Modern *omics* experiments pertain not only to the measurement of many variables but also follow complex experimental designs where many factors are manipulated at the same time. This data can be conveniently analyzed using multivariate tools like ANOVA-simultaneous component analysis (ASCA) which allows interpretation of the variation induced by the different factors in a principal component analysis fashion. However, while in general only a subset of the measured variables may be related to the problem studied, all variables contribute to the final model and this may hamper interpretation.

**Objectives:**

We introduce here a sparse implementation of ASCA termed group-wise ANOVA-simultaneous component analysis (GASCA) with the aim of obtaining models that are easier to interpret.

**Methods:**

GASCA is based on the concept of group-wise sparsity introduced in group-wise principal components analysis where structure to impose sparsity is defined in terms of groups of correlated variables found in the correlation matrices calculated from the effect matrices.

**Results:**

The GASCA model, containing only selected subsets of the original variables, is easier to interpret and describes relevant biological processes.

**Conclusions:**

GASCA is applicable to any kind of *omics* data obtained through designed experiments such as, but not limited to, metabolomic, proteomic and gene expression data.

## Introduction

In systems biology and functional genomics designed experiments are nowadays very common: this refers to research situations in which a dependent variable *x*, measured on a (biological) system, constitutes the response to *I* independent variables, called factors (or treatments), whose levels are controlled by the experimenter. The variety of designed experiments ranges from simple case–control settings to complex scenarios where many factors are manipulated simultaneously.

When a measured variable is a function of (several) factors, analysis of variance (ANOVA) is a well establish technique to analyze the data (Searle and Gruber 2016). However, in functional genomics and systems biology many variables ($x_1, x_2,\ldots ,x_J$) are usually measured on a system, like in metabolomics, proteomics and transcriptomics experiments where hundreds to thousands of variables are acquired. In these cases a single ANOVA model can be fitted separately on each variable. Although effective (this is what is usually done in the case of gene expression data), this approach does not take into account the relationships existing among the variables, i.e. the multivariate nature of the problem is discarded (Saccenti et al. 2014). Because biological variables, such as metabolites or genes, are often interrelated, it is desirable, in many occasions, to analyze all the *J* variables simultaneously (Saccenti et al. 2014). ANOVA can be generalized to the multivariate case through multivariate-ANOVA (MANOVA, see Eq. 1) (Bibby et al. 1979). In contrast with running several separated ANOVAs, MANOVA takes into account the correlation among the $x_1, x_2,\ldots ,x_J$ dependent variables when testing for the significance of the factor effects; moreover, since all variables are used simultaneously it also reduces the risk of Type I error (O’Brien and Kaiser 1985).

Unfortunately, in the case of high dimensional *omics* data the MANOVA model often breaks down because the number of variables *J* is larger than the number of observations *N*, and this results in the covariance matrices involved in the calculations for significance testing and data visualization to be singular, which makes the MANOVA solution non achievable. A classical remedy to this problem is regularization which involves numerical manipulation of the covariances matrices to remove singularity: this is the so-called regularized-MANOVA for which several solutions have been proposed (Engel et al. 2015; Ullah and Jones 2015). Other solutions regard non-parametric reformulations of the problem (Legendre and Anderson 1999; Anderson 2001).

A different approach is to combine ANOVA with principal component analysis (PCA) to reduce the dimensionality of the data to be analyzed. Earlier applications involved performing MANOVA on a reduced data set consisting of the low dimensional scores of a PCA model fitted to the data (Bratchell 1989). However this approach suffers from the limitation that PCA is not able to resolve the different type of variation induced by the different factors which may be confounded by the initial PCA model. These limitations can be overcome by using ANOVA-simultaneous component analysis (ASCA) (Smilde et al. 2005; Jansen et al. 2005).

ASCA uses an ANOVA model to first decompose the data matrix into factor effect (and interaction) matrices containing the average values for each factor level (and interaction thereof); then a PCA model is fitted separately on each effect matrix to extract and assess the contribution of each variable to the systematic variability induced by each experimental factor. Hence, an ASCA model can be explored and interpreted like a standard PCA model. A similar but less powerful approach is ANOVA-PCA (Harrington et al. 2005; Zwanenburg et al. 2011).

While ASCA retains both the flexibility of the ANOVA framework to account for (possibly) very complicated experimental designs and the versatility of PCA as a data dimensionality reduction method, it also inherits the limitations of the classical principal component analysis.

PCA is a valuable tool for data reduction and exploration: however since it is essentially a data factorization based on variance maximization, it presents two main shortcomings when data understanding and interpretation are the goal of the analysis. First, it cannot distinguish between variance which is unique for a single variable and variance which is shared among several variables and this can seriously hamper the unveiling of (possibly) hidden relationships existing among variables (Jolliffe 2002). Second, the principal components are linear combinations of all the variables simultaneously and this greatly complicates data interpretation since all variables contribute to the PCA model (Jolliffe et al. 2003).

While the first limitation can be addressed by using methods that focus on shared variance, like Factor analysis (Fabrigar et al. 1999), better interpretability of the PCA solution can be obtained by imposing a simple structure on the components, in such a way that the components are combinations of a smaller number of original variables. This is the realm of sparse methods and many formulations have been proposed (see for instance sparse implementations using LASSO (Jolliffe et al. 2003; Zou et al. 2006), group LASSO (Jacob et al. 2009) or structure-based regularization criteria (Jenatton et al. 2009).)

We recently proposed a new sparse implementation of PCA where sparsity is defined in terms of groups of (correlated) variables identified from the data to be analyzed, called group-wise PCA (GPCA) (Camacho et al. 2017). The GPCA solution is such that every principal component contains loadings different from zero only for a group of variables. This grounds on the framework of simplivariate models (Hageman et al. 2008; Saccenti et al. 2011) which aim to retain both the comprehensiveness of a multivariate model and the simplicity of interpretation of a univariate one, under the assumption that a given (biological) phenomenon may not be accounted by all measured variables but only by one, or more, subsets of variables. This kind of sparsity is natural in biological problems: examples are sets of metabolites participating in the same metabolic network or co-expressed and co-regulated genes which are expected to exhibit a correlative behavior. Thus, the sparsity exploited in GPCA is different from the one used in sparse PCA implementations based on regularization: in the latter sparsity is obtained by forcing to zero the loadings corresponding to some variables by controlling one or more regularization parameters which must be algorithmically optimized. In GPCA the parameter controlling sparsity is immediately related to the strength of association among (groups of) variables as expressed, for instance, by their correlation. In addition the relationship between the threshold on the correlation value and the level of sparsity, i.e. the size and the number of groups selected, can be graphically visualized and explored, consistently with a data exploratory philosophy.

In this paper we propose to replace the PCA step in ASCA with GPCA, arriving to a group-wise sparse version of ASCA termed Group-wise ANOVA-simultaneous component analysis (GASCA). The aim is to improve the interpretability of the ASCA solution when analyzing complex data sets. The characteristics of this approach are illustrated with simulations and by comparing the GASCA model with both PCA and the original ASCA and by the analysis of a designed plant and human metabolomics experiments. The paper is organized as follows: The Sect. 2 introduces the ANOVA–MANOVA framework and details the mathematics and the properties of the PCA, GPCA, ASCA and GASCA models. The Sect. 3 presents the data description and details on software used. Finally, Results and discussion of PCA, ASCA and GASCA modeling of simulated and experimental data are given: in particular, the fitting of the GASCA model is illustrated step by step using real experimental data. Some final considerations are offered in the Sect. 5.

## Theory

### (M)ANOVA model

We consider here a study design involving two factors $\alpha$ and $\beta$ with *A* and *B* levels, respectively, where *J* variables are measured. For a balanced design, in which every measurement is replicated *R* times for each combination of factor levels, there are in total $N=ABR$ observations. The multivariate ANOVA model (MANOVA) is given by

1 $$\begin{aligned} \mathbf {X}= \mathbf {1}\mathbf {m}^{\mathrm {T}} + \mathbf {X}_\alpha + \mathbf {X}_\beta + \mathbf {X}_{(\alpha \beta )} + \mathbf {E}\end{aligned}$$

where the first term $\mathbf {1}\mathbf {m}^{\mathrm {T}}$ represents the overall mean for the data, $\mathbf {1}$ is a column vector of ones of length *N* and $\mathbf {m}^\mathrm{T}$ is a row vector of size *J* with the averages over the data for each variable. The effect matrices $\mathbf {X}_\alpha$ and $\mathbf {X}_\beta$ contain the level averages for each factor and the $\mathbf {X}_{(\alpha \beta )}$ describes the interaction between the two factors. The variation that cannot be represented by the model is collected in the residual matrix $\mathbf {E}$. Equation (1) is the starting point of both ASCA and the newly proposed GASCA.

### The PCA model

Given a data matrix $\mathbf {X}$ of size $N \times J$ (observations $\times$ variables), the standard PCA model follows the expression:

2 $$\begin{aligned} \mathbf {X} = \mathbf {T}_{H}\mathbf {P}_{H}^\mathrm {T} + \mathbf {E}_H, \end{aligned}$$

where $\mathbf {T}_{H}$ is the $N \times H$ score matrix containing the projection of the objects onto the *H* principal components subspace, $\mathbf {P}_{H}$ is the $J \times H$ loading matrix containing the linear combination of the variables represented in each principal component, and $\mathbf {E}_H$ is the $N \times J$ matrix of the residuals. Usually *H* is chosen to be much smaller than *J*.

### The group-wise PCA model

The Group-wise Principal component analysis (GPCA) (Camacho et al. 2017) is a sparse formulation of the PCA algorithm where sparsity is defined in terms of groups of correlated variables: every component contains non-zero loadings for a single group of correlated variables which simplifies the interpretation of the model. The GPCA approach consists of three steps:

1. Computation the association map $\mathbf {M}$ form the data
2. Identification of the groups of associated variables
3. Calibration and fitting of the GPCA model

The GPCA modeling starts with the definition of a $J \times J$ association map $\mathbf {M}$ computed from the data and describing the relationship among the variables. In the original formulation of GPCA (Camacho et al. 2017) the MEDA approach (Missing-data for Exploratory Data analysis) (Camacho 2011) was used to define $\mathbf {M}$. Briefly, MEDA consists of a post-processing step after the PCA factorization to infer the relationships among variables using missing data imputation (Arteaga and Ferrer 2002; Arteaga and Ferrer 2005). The *l*, *j*-th element $m_{lj}$ (for variables $l = 1,\ldots ,J$ and $j = 1,\ldots ,J$) of the MEDA map $\mathbf {M}$ can be expressed as (Arteaga, 2011):

3 $$\begin{aligned} m_{lj} = \dfrac{ \left\{ \mathbf x _l^\mathrm {T}\mathbf x _j + (\mathbf e ^Q_l)^\mathrm {T}\mathbf e ^Q_j\right\} \cdot \mathrm {abs}\left\{ \mathbf x _l^\mathrm {T}\mathbf x _j - (\mathbf e ^Q_l)^\mathrm {T}\mathbf e ^Q_j\right\} }{\sigma _\mathbf{x _l}^2\sigma _\mathbf{x _j}^2} \end{aligned}$$

where $\mathbf {e}^Q_l$ is the vector of residuals for the *l*-th variable in the PCA model with *Q* latent variables; data is assumed to be centered. Practically, this approach uses a missing data strategy to estimate the correlation between any two variables: this approach has been found to be effective in filtering out noise when estimating correlations (Camacho 2010). Here we set *Q* using the *ckf* cross-validation algorithm (Saccenti and Camacho 2015b) as in the original GPCA formulation (Camacho et al. 2017) but other approaches are possible (Saccenti and Camacho 2015a). Note that if we set $Q = \mathrm {rank}(X)$ the MEDA map from Eq. (3) reduces to a standard Pearson correlation map where the original magnitudes are replaced by their squared values while the sign is retained. In place of the MEDA map any square symmetric matrix describing mutual relationship among the variables can be used as an input for GPCA (like mutual information, as often done in the case of gene expression data): since metabolomic data is usually analyzed in term of correlations (Saccenti et al. 2014; Saccenti 2016) we will present also a GASCA implementation based on correlations. Because relationships among metabolites cannot be assumed to be linear, we use here Spearman’s rank correlation, which equal to the Pearson’s correlation of the ranks of the variables. In addition, to reduce the risk of including chance associations we force to 0 correlations for which the associated *P* value is larger than 0.01. Summarizing the elements $m_{lj}$ of the association map $\mathbf {M}$ based on correlations are:

4 $$\begin{aligned} m_{lj}= {\left\{ \begin{array}{ll} r_{lj} &{} \text {if}\ P\!\mathrm {-val}\le 0.01 \\ 0 &{} \text {otherwise} \end{array}\right. } \end{aligned}$$

Once the association map $\mathbf {M}$ is defined, the *K* groups of correlated/associated variables are identified using the so called group identification algorithm (GIA). Briefly, the GIA works as follows: given $m_{lj} \in [-1, 1]$ the *l*, *j*-th element of $\mathbf {M}$, and $|\gamma |<1$, the group $S_k$ is built in such a way that $|m_{lj}|> \gamma$ for all *l*, *j* in $S_k$ with maximum cardinality. The *j*-th variable is not in group $S_k$ when $|m_{lj}| \le \gamma$ with at least one variable *l*-th in $S_k$. The GIA algorithm is fully detailed in the Appendix of reference (Camacho et al. 2017). As in the case of the definition of the association map $\mathbf {M}$, other strategies can be implemented to define the $S_k$ groups, such as hierarchical clustering (Langfelder et al. 2007), which can be a convenient approach when dealing with high-dimensional genomic data. The smallest possible size for $S_k$ is 1: however since the goal is to identify groups of correlated variables and not singletons or pairs, the minimal groups size can be user-defined. Here we set this to be equal to $\sqrt{J}$, where *J* is the total number of variables; this is a longstanding common choice in statistics and machine learning practice to select the minimum size of subset of variables (Summerfield and Lubin 1951; Guyon and Elisseeff 2003).

The GIA is an ordination algorithm and takes as input precomputed correlations, hence its performance is not affected by the noise in the data (only the estimation of correlation is) nor by the number of variables considered, although the computational cost increases with the number of variables. We remark that (group-wise) sparsity is a property of the data and not of the method used for the analysis of data. The GPCA approach has the advantage that situations where sparse modeling is not suitable can be easily detected through the correlation (association) map $\mathbf {M}$: in absence of correlation structure or when the *S* groups contain mostly singletons or very few variables GPCA (and GASCA) are not recommended to analyze the data.

Once the $S_k$ have been defined, the GPCA algorithm first computes *K* candidate loading vectors, where the *k*-th loading vector has non zero elements associated to the variables in the *k*-th group $S_k$ (the loadings for variables which are not in $S_k$ are then set to zero.) From these, only the loading with the largest explained variance is retained in the model and it is used to deflate data matrix $\mathbf {X}$. The complete GPCA algorithm is outlined in the Appendix.

The parameter $\gamma$ is user defined and can be determined by visually inspecting the correlation or the MEDA map $\mathbf {M}$ (or any other association map) and the output of the GIA, since $\gamma$ simultaneously controls both the size and the number of groups of correlated variables. This approach is consistent with the exploratory data analysis philosophy under with the group-wise PCA was developed. Moreover, $\gamma$ has a direct interpretation as a threshold on the strength of the correlation, and it may be easier to tune than the regularization parameters that characterize other sparse implementations of PCA. In the Sect. 4 we guide the reader through the selection process of the $\gamma$ parameter during the analysis of simulated and experimental data.

### The ANOVA simultaneous components model

ANOVA-simultaneous component analysis (ASCA) (Smilde et al. 2005) aims to overcome the limitations of MANOVA reducing the original number *J* of variables in the effect (interaction) matrices $\mathbf {X}_{i}$ by replacing them with a lower number ($H<<J$) of principal components. In this way it is possible to explore the relationship among variables and their contribution to variability observed in the data even in the case of singular data covariance matrices. This is accomplished by fitting a PCA model (see Eq. 2) to each of the effect (interaction) matrices $\mathbf {X}_{i}$ in the model given by Eq. (1). The ASCA decomposition in principal components is given by

5 $$\begin{aligned} \mathbf {X}_i = \mathbf {T}_i^{\mathrm {PCA}}\left( \mathbf {P}_i^{\mathrm {PCA}}\right) ^\mathrm {T}\quad \quad i \in \{\alpha , \beta , \alpha \beta \} \end{aligned}$$

where $\mathbf {T}_i^{\mathrm {PCA}}$ and $\mathbf {P}_i^{\mathrm {PCA}}$ are the scores and loadings matrices of a PCA model fitted on the effect (interaction) matrix *i*.

Note that here we have dropped for convenience the subscript *H* referring to the dimensionality of the PCA model as given in Eq. (2). It is intended that *H* components are retained to fit the ASCA model and that the number of components used can be different for different effect matrices (thus $H = H_i$).

Since the PCA models are fitted to the effect matrices which contain the averages of variables within the same factor levels, the variation between replicates in each level is lost. However this information can be retrieved by projecting the effect matrix ($\mathbf {X}_i$) plus the residual matrix ($\mathbf {E}$) onto the space defined by the loading matrix $\mathbf {P}_i$ for the PCA model for $\mathbf {X}_i$ as first proposed by Zwanenburg *at al.*(Zwanenburg et al. 2011):

6 $$\begin{aligned} \mathbf {Y}_{i}=(\mathbf {X}_{i}+\mathbf {E})\mathbf {P}_{i}^\mathrm {PCA}= \mathbf {T}_{i}^\mathrm {PCA}+\mathbf {E}\mathbf {P}_{i}^\mathrm {PCA}\quad \quad i\in \{\alpha ,\beta ,\alpha \beta \}. \end{aligned}$$

The projection $\mathbf {Y}_{i}$ represents the variability of the replicates in terms of the loadings $\mathbf {P}_{i}$ of the PCA model for $\mathbf {X}_{i}$.

*Important note*: for simplicity of illustration we show here a 2-way ASCA model but ASCA can be applied to designed experiments with an arbitrary number of factors and levels.

### The group-wise ANOVA simultaneous component analysis

While ASCA is well suited for analyzing designed experiments, the ASCA model may be complicated to interpret since the principal components are linear combinations of all the variables: to overcome this limitation we propose here to replace the PCA step in ASCA with GPCA to arrive at an ASCA solution which is sparse in a group-wise sense.

With respect to the 2-way model considered in Eq. (1) the GASCA model assumes the form

7 $$\begin{aligned} \mathbf {X}_i = \mathbf {T}^{\mathrm {GPCA}}_i\left( {\mathbf {P}^{\mathrm {GPCA}}_i}\right) ^\mathrm{T} \quad \quad i \in \{\alpha , \beta , \alpha \beta \} \end{aligned}$$

where $\mathbf {T}^{\mathrm {GPCA}}_i$ and $\mathbf {P}^{\mathrm {GPCA}}_i$ are the scores and loadings matrices for the *i*-th factor effect (or factor interaction matrix) obtained using GPCA (see Eqs. (12) and (11) in the Appendix). Operatively, a group-wise ASCA consists of three steps:

1. Definition of the correlation/association maps (matrices) $\mathbf {M}_i$, one for each effect and interaction matrices
2. Setting the convenient $\gamma$ parameter to define the number and the size of the $S_1, S_2,\ldots ,S_k$ groups of correlated variable for each maps
3. Fitting of the GASCA model to obtain one set of loadings for each effect and interaction matrices.

We consider here two approaches to define the association maps $\mathbf {M}_i$. The first is to derive $\mathbf {M}_i$ starting from correlation (or any other association measure) calculated from the effect matrices $\mathbf {X}_i$ which are intrinsically low noise: this is fully consistent with the ASCA framework. An alternative approach is to obtain $\mathbf {M}_i$ from the sum of the factor (and interaction) matrices and residual matrix. As an example, for factor $\alpha$ in the two-way MANOVA design from Eq. (1), $\mathbf {M}_\alpha$ can be obtained from

8 $$\begin{aligned} \mathbf {E}_\alpha = \mathbf {X}- \mathbf {1}\mathbf {m}^{\mathrm {T}} - \mathbf {X}_\beta - \mathbf {X}_{(\alpha \beta )} \end{aligned}$$

Note that this is the same matrix used to calculate the scores. When this method is used, the MEDA approach is better suited to define association among variables than standard correlations. This approach should be used in the case of a design with factors with two levels. This is because with two levels, the correlation cannot be computed from the effect matrix $\mathbf {X}_i$, since this will always result in a matrix containing only − 1 and 1 values arising from the design and not from the biology of the data. In the Sect. 4 simulated and real data are analyzed using both approaches and which method to use depends on the nature of the data.

Finally, these three steps should be preceded by a statistical validation of the multivariate effect: this is illustrated in the next section. It should be noted that when the design is not balanced (i.e. when there is not the same number of observations for each factor level) the effects estimates are not orthogonal and fitting the model becomes cumbersome and requires *ad-hoc* approaches (Rawlings et al. 2001). The ASCA framework has been extended to work with unbalanced design (Thiel et al. 2017); an equivalent approach can be used for GASCA. For the sake of simplicity GASCA has been illustrated with a 2-way ANOVA design, but it is generalizable to any number of factors and levels and the software code provided (see Sect. 3.3) will work with a general *N*-way design.

### Validation of multivariate effects

Since GASCA is designed to obtain sparse models of the effect matrices obtained from designed *omics* experiments, it is necessary, before fitting a GASCA (or an ASCA) model, to validate whether the levels observed in the sample reflect effects specific in the population or originate by sampling fluctuations. This problem has been addressed in the ASCA context (Vis et al. 2007) and the solution proposed transfers directly to the GASCA case. Following (Vis et al. 2007) we employ a permutation approach to assess the statistical significance of the high-dimensional effects observed in GASCA since the standard MANOVA approach based on the multivariate extension *F*-test can not be applied in this framework because the number of variables is larger than the number of samples. The permutation approach has several advantages: it is optimal for small data sets, is free of distributional assumptions, and gives exact probability values (Berry et al. 2016).

The procedure validates the ANOVA partitioning of the data and should be performed before fitting the GASCA model to the data since it makes no sense to fit a model to effect (interaction) matrices which do not contain significant factor effects but are likely to contain sampling and/or measurement noise.

## Material and methods

### Experimental plant data

#### Experimental design

This data set contains the time-resolved metabolomic response of *Arabidopsis thaliana* towards changing light and/or temperature (Caldana et al. 2011). The original data comprises both metabolomic and transcriptomic data measured under four different light conditions (D: dark, LL: low light, L: light, and HL: high light) at three different temperatures ($4{}^{\circ }$C, $21{}^{\circ }$C, $32{}^{\circ }$C) with different growth time from 0 to 360 min for a total of 19 time points. We consider here only data acquired at $21{}^{\circ }$ and at time points (t = 0, 5, 10, 20, 40, 80, 160 min) under the four light conditions. The data here analyzed has a design with two factors (light condition and time) with 7 and 4 levels, respectively. The complete data is available through the original publication (Caldana et al. 2011).

#### Missing data imputation

There were 147 missing values in the data set: since removing observations with a missing value would drastically reduce the number of observations, we imputed the missing values replacing them with the average value of the cell and adding a random number drawn from a normal distribution with 0 mean and variance equal to the data cell variance.

#### Data cleaning

There are six biological replicates for each factor level, except for the LL level which has only 5. Since a balanced design is need for both ASCA and GASCA, we randomly removed 1 observation from the factors with 6 replicates. Data for starting condition ($t = 0$ min) was given once only for level *L* of the light condition factor and was replicated for all the remaining level (hence, the data for first time point is identical for all light conditions) to remove imbalance. Two variables (raffinose and glycine) were removed from the data set because we suspected that something went wrong with the measurement and/or the quantification (data analyzed using PCA): several observations where characterized by disproportionately high values (up to 2 order of magnitudes) for these metabolites which were discarded. A problem with the measurement of glycerol for light condition L and $t = 10$ min was also detected. The value was replaced with the cell average. After this correction no more outliers were evident. The final data matrix has dimensions $140(= 4\times 7\times 5)\times 67$.

#### Data pre-processing

Metabolite abundances were normalized by dividing each raw value by the median of all measurements of the experiment for one metabolite.

#### Experimental details

For convenience of the reader we give a short summary of the experimental setup. We refer to the original publication (Caldana et al. 2011) for more details. Plants grown at 21 °C with a light intensity of 150 µE × m^−2^ × s^−1^ were either kept at this condition or transferred into seven different environments (4 °C, darkness; $21{}^{\circ }$, darkness;  32 °C, darkness; 4 °C, 85 µE × m^−2^ × s^−1^; 21 °C, 75 µE × m^−2^ × s^−1^; 21 °C, 300 µE × m^−2^ × s^−1^; 32 °C, 150 µE × m^−2^ × s^−1^.

Metabolites were extracted from single rosettes in a total of six replicates. Extraction and derivatization of metabolites from leaves using GC–MS were performed as previously reported (Lisec et al. 2006). GC–MS data were acquired on a Agilent 7683 series autosampler coupled to an Agilent 6890 gas chromatograph Leco Pegasus two time-of-flight mass spectrometer; acquisition parameters were as reported in (Weckwerth et al. 2004). Peak detection, retention time alignment and library matching were obtained using the TargetSearch package (Cuadros-Inostroza et al. 2009).

### Experimental human data

#### Experimental design

We randomly selected two subjects from the METREF study (Assfalg et al. 2008) where 22 healthy subjects were sampled for their urinary profile on  40 consecutive days. The data has a 1-way ANOVA design with two level (Subject 1 and Subject 2). The data is available through the KODAMA R package (Cacciatore et al. 2017).

#### Data pre-processing

Bucketing was applied to the NMR spectra after the removal of region with $\delta> 9.5$ ppm, $4.5<\delta < 6.0$ppm, and $\delta < 0.5$ ppm, containing water and urea signals. each spectrum was divided into sequential bins of 0.02 ppm width, which were integrated using AMIX software (Bruker BioSpin). Finally, total area normalization was carried out on all the spectra. Further, bins corresponding to noise and empty spectral areas were removed to reduce dimensionality. To make the design balanced, 37 spectra for each subject were considered: the final dataset has size $74\times 206$.

#### Experimental details

$^{1}$H NMR spectra were acquired using a Bruker 600 MHz metabolic profiler (Bruker BioSpin) operating at 600.13 MHz proton Larmor frequency and equipped with a 5 mm CPTCI cryoprobe. Each urine sample was acquired with a NOESY-presaturation pulse sequence. Details on sample preparation and further information on the NMR experimental setup can be found in the original publication (Assfalg et al. 2008) and in other publications where the data has been analyzed (Bernini et al. 2009; Ghini et al. 2015).

### Software

The GPCA, ASCA and GASCA and GIA algorithms are freely available in the Matlab MEDA toolbox (Camacho et al. 2015) at the address: github.com/josecamachop/MEDA-Toolbox. The GASCA code is based on the original Matlab code for ASCA by G. Zwanenbourg (Zwanenburg et al. 2011). The function to call is gasca: typing help gasca in the Matlab command windows will prompt instructions and a worked out example to perform GASCA.

## Results and discussion

### Simulations

We begin presenting the GASCA of a simple simulated data set to show how GASCA models data which is sparse in a group-wise fashion: the data follows a two factor design ($\alpha$ and $\beta$), with four and three levels, respectively, and no interaction, with one group of correlated variables contributing only to the first factor, and another group of variables contributing only to the second factor; both groups consists of five variables. There are 100 observations and 50 variables; the two factors are both significant at the 0.01 level.

**Fig. 1:**
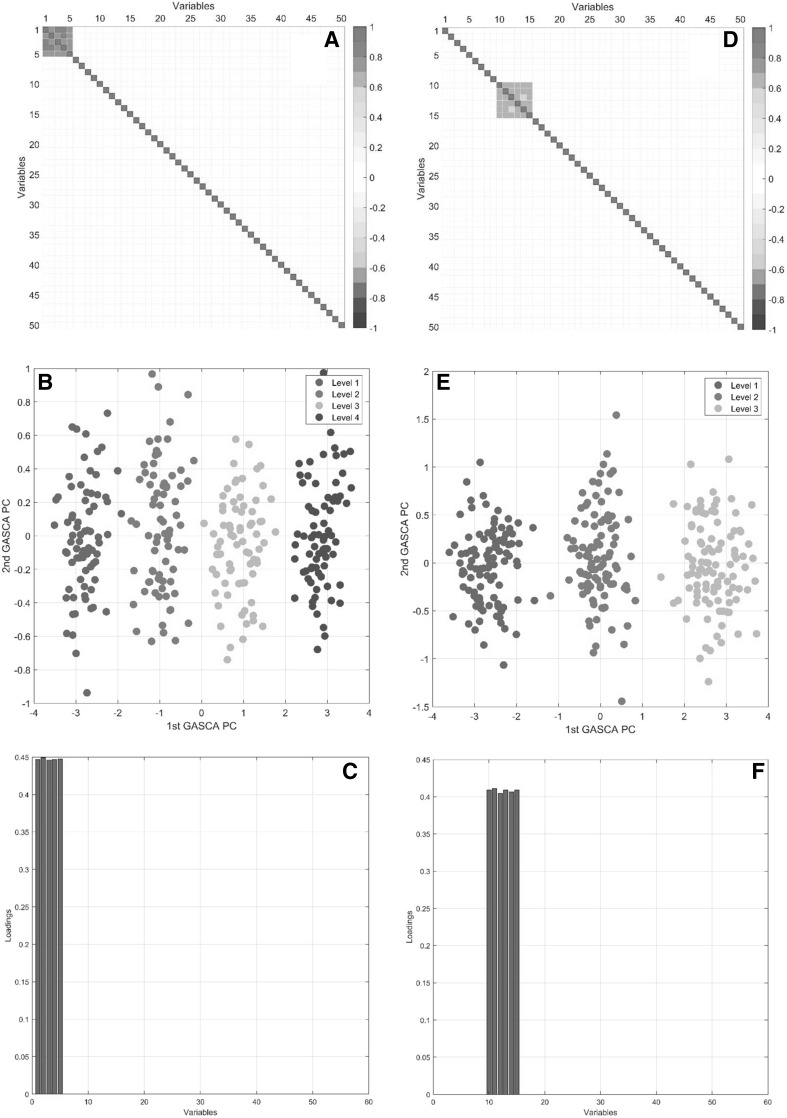
GASCA modeling for the first factor of the simulated examples: **a** MEDA map built from the residuals; **b** score plot **c** loadings. GASCA modeling for the second factor of the simulated examples: **d** MEDA map built from the residuals; **e** score plot and **f** loadings

The analysis starts with the construction of the association matrices $\mathbf {M}_\alpha$ and $\mathbf {M}_\beta$ for the effect matrices $\mathbf {X}_\alpha$ and $\mathbf {X}_\beta$. As discussed in the Sect. 2.3, there are several strategies to construct such matrices: for this example we build MEDA maps (see Eq. 3) starting from the matrices $\mathbf {E}_\alpha$ and $\mathbf {E}_\beta$ defined in Eq. (8). Another approach, based on correlations, will be shown in the analysis of the plant metabolomics data (see Sect. 4.4). The MEDA maps for the two factor are shown in Fig. 1 panels a and d, respectively. The two groups of associated variables are evident: the threshold $\gamma$ controlling the sparsity of the solution can be chosen by inspecting the MEDA maps and we set $\gamma = 0.8$ for both factors. This is a rather straightforward situation; association maps for real data, especially for metabolomics data, are usually more complicate: a guided procedure to select $\gamma$ will be shown in Sect. 4.4. The score plots resulting GASCA models for the two factors are shown in Fig. 1 in panel b and e, while the loadings are given in panels c and f. The GASCA solution is sparse in a group-wise fashion, with just one group of variables contributing to each factor, which greatly facilitate interpretation, correctly retrieved by the model.

### PCA modeling of the plant data

**Fig. 2:**
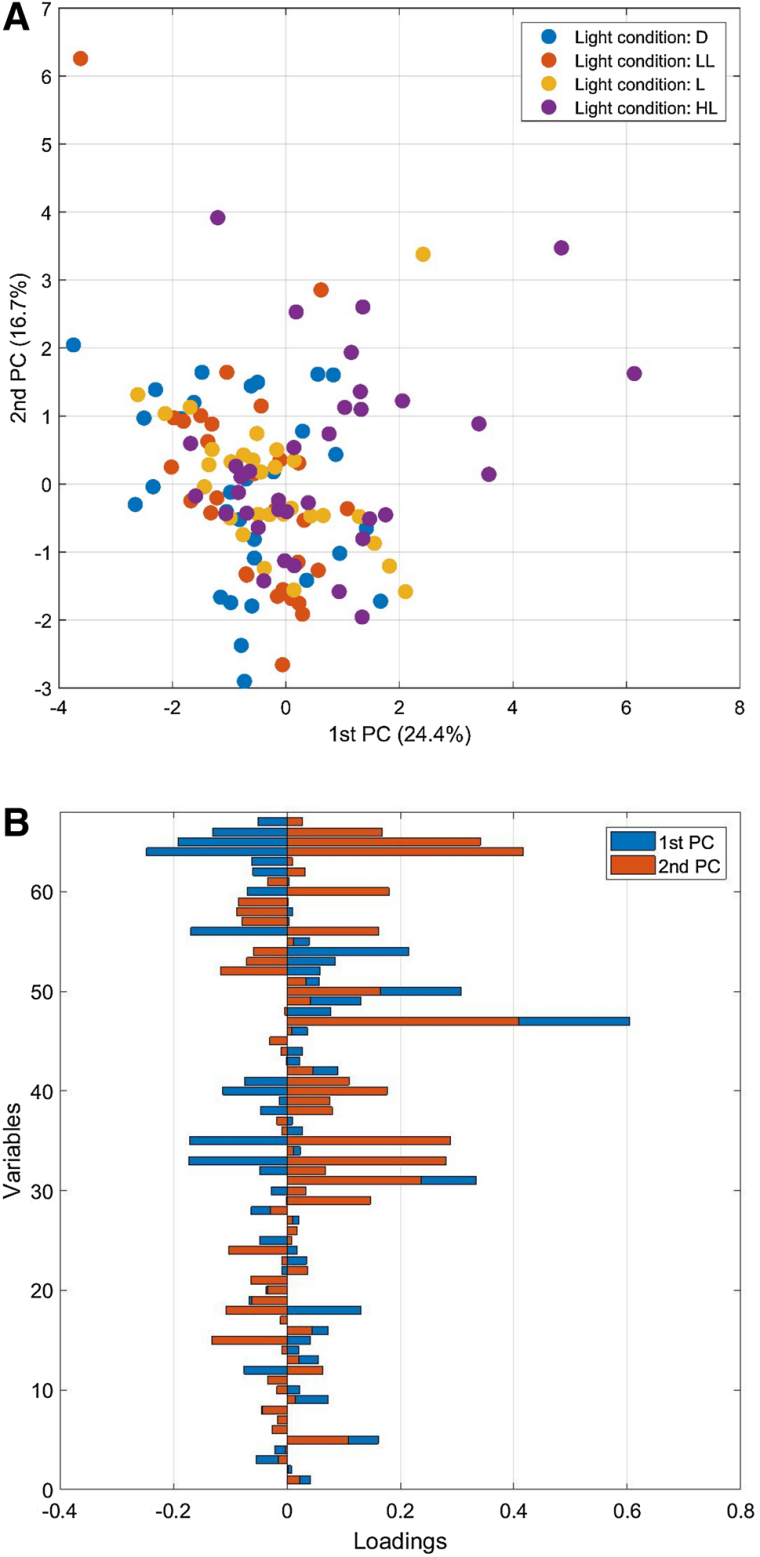
PCA model for the *Arabidopsis* data: **a** scores and **b** loadings. Only factor 1 (light condition) is color coded. The levels for factor 1 are: dark (D), light (L), low light (LL) and high light (HL)

In the following we present a comparison of PCA, ASCA and GASCA models obtained on a designed plant metabolomic experiment. We begin by fitting a standard PCA model (see Eq. 2) to the data. The scatter plot of the first two principal components and the corresponding loading vectors are given in Fig. 2. It appears that a simple PCA is not well suited for analyzing this data since it does not distinguish between the groups in the data: factors and levels are mixed up in the score plot. Moreover, loadings are complicate to interpret since all variables contribute to the final model.

### ASCA modeling of the plant data

Before applying ASCA (and, of course, GASCA) we test the significance of effects for the two factors of the experimental design (light conditions and time) and their interaction. Applying a permutation test with $n_{perm} = 10^4$ to test the significance of the factors the calculated *P* values are 0.0001, 0.0001 and 0.0278 for light condition, Time and their interaction, respectively. Since all factors and interactions are significant we will fit the ASCA (and later GASCA) model on all effect and interaction matrices, *i.e* $\mathbf {X}_{\alpha }$, $\mathbf {X}_{\beta }$ and $\mathbf {X}_{\alpha \beta }$.

The *Arabidopsis* data follows a two factors design (time and light condition) with 4 and 7 levels respectively, thus there are two matrices for the effects ($\mathbf {X}_{\alpha }$ and $\mathbf {X}_{\beta }$ and one matrix for the interaction $\mathbf {X}_{\alpha \beta }$.) The overall mean explains the $86.7\%$ of the total sum of squares, the two factors $0.86\%$ and $1.3\%$, the interaction $2.1\%$ and the residuals $9.1\%$.

**Fig. 3:**
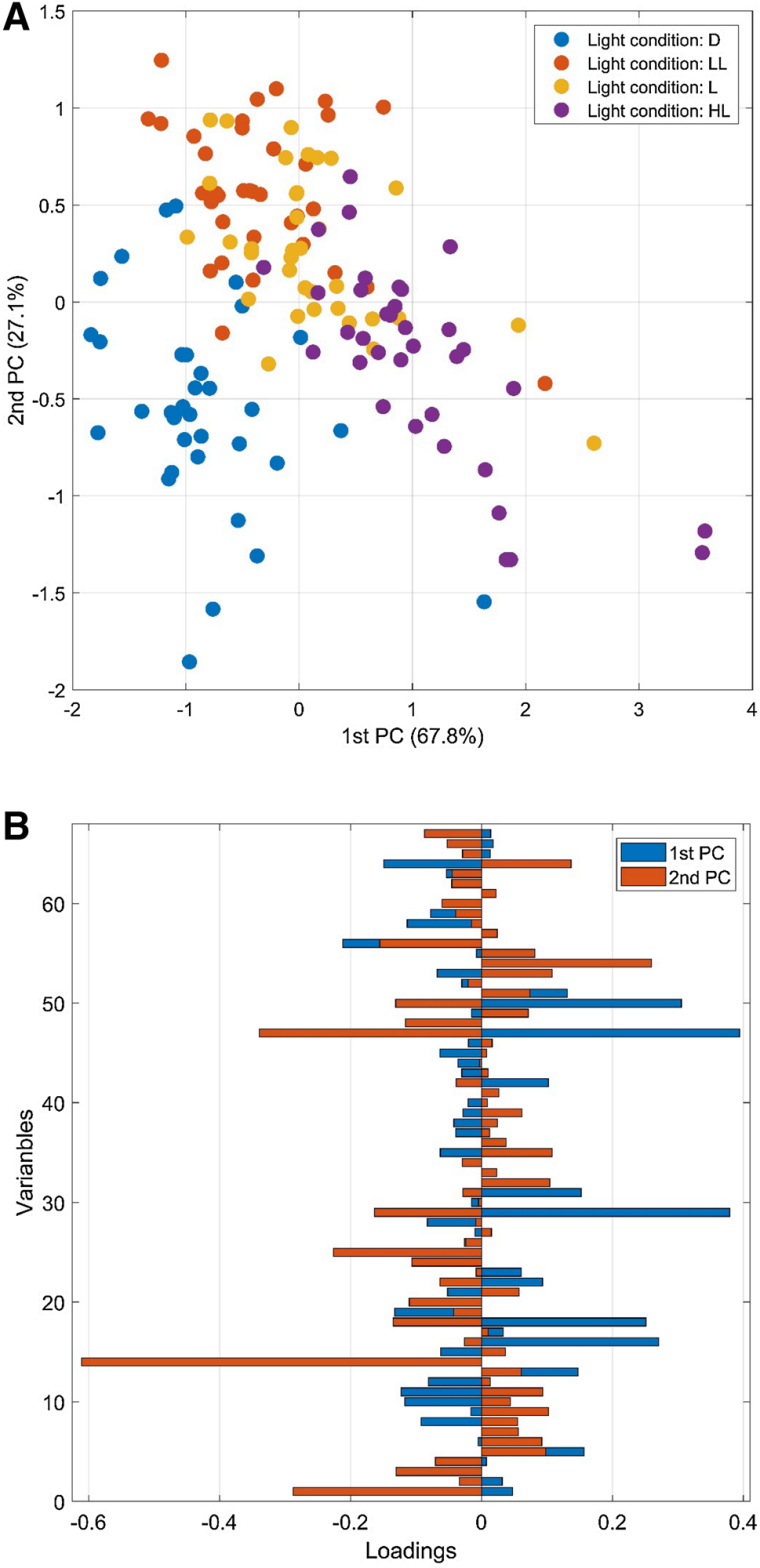
ASCA model for the *Arabidopsis* data: **a** scores and **b** loadings. Only factor 1 (light condition) is color coded. The levels for factor 1 are: dark (D), light (L), low light (LL) and high light (HL)

A scatter plot of the first two ASCA components and the corresponding loading vectors are given in Fig. 3 for the factor 1 (light conditions); ASCA is able to resolve the different levels of the treatment. but the interpretation is not straightforward. As almost all metabolites contribute to the model (i.e. have non zero loadings), this makes hard to identify which metabolites are important to explain the systematic variation induced on the system by manipulating the light condition. In the next section we show how GASCA can simplify data understanding and interpretation.

### Group-wise ASCA modeling of the plant data set

Once the significance of the effects is assessed (this has been already done in the ASCA modeling showed in the previous section), the group-wise ASCA modeling starts from the construction of the variable association map $\mathbf {M}$ from the original data. Since there is one GASCA model for each effect matrix and for each interaction (see model in Eq. 1) there are three maps ($\mathbf {M}_i$ with $i \in \{\alpha , \beta , \alpha \beta \}$) to be built. As detailed in the Sect. 2 we use Spearman correlation to quantify the relationships among the metabolites, retaining only those which are statistically significant (see Eq. 4)

**Fig. 4:**
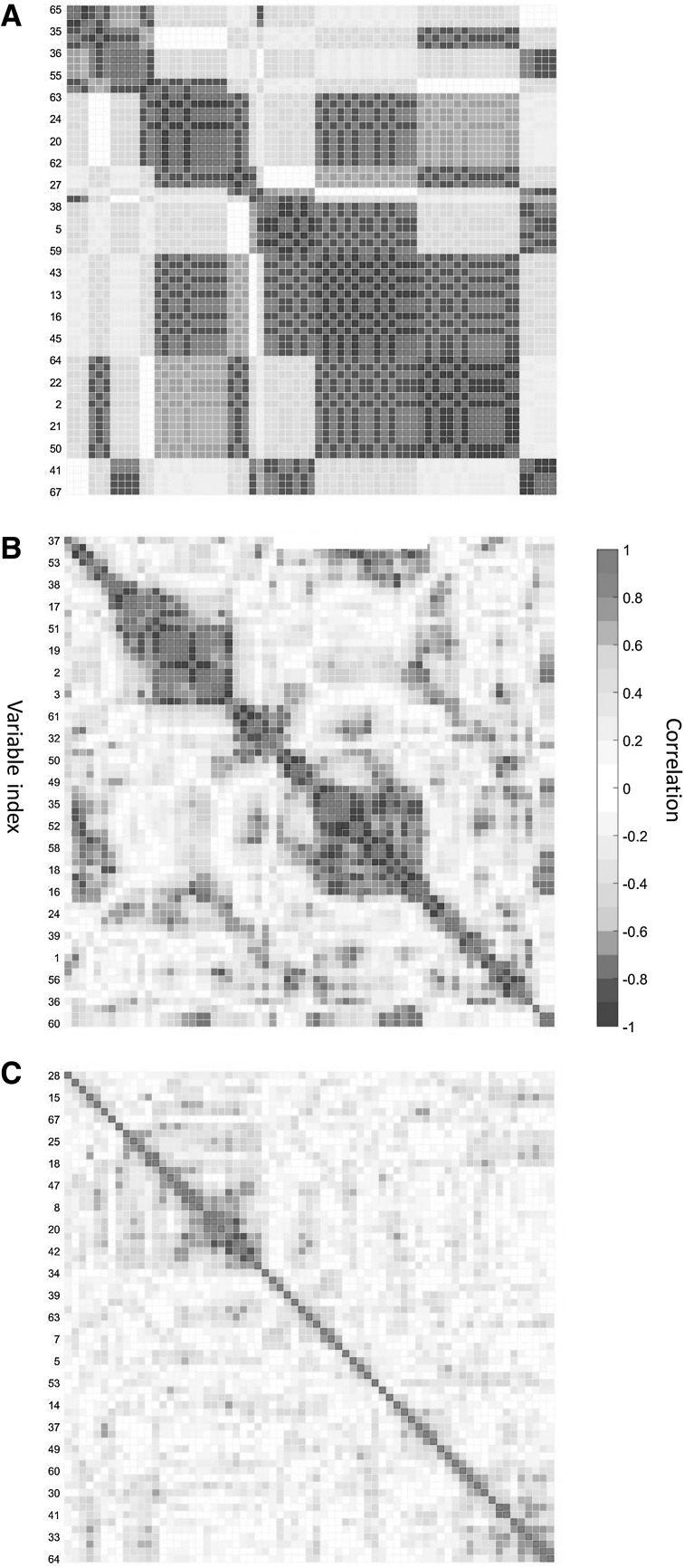
MEDA maps for **a** factor 1, light condition; **b** factor 2, iime; **c** interaction light$\times$Time

The Spearman correlation maps $\mathbf {M}_{i}$ are shown in Fig. 4. Several groups of (highly) correlated variables are evident in all three cases; however there are many variables not contributing to the correlation structure of the data. This is a situation in which a GPCA implementation in ASCA is adequate because data is sparse in a group-wise fashion

**Fig. 5:**
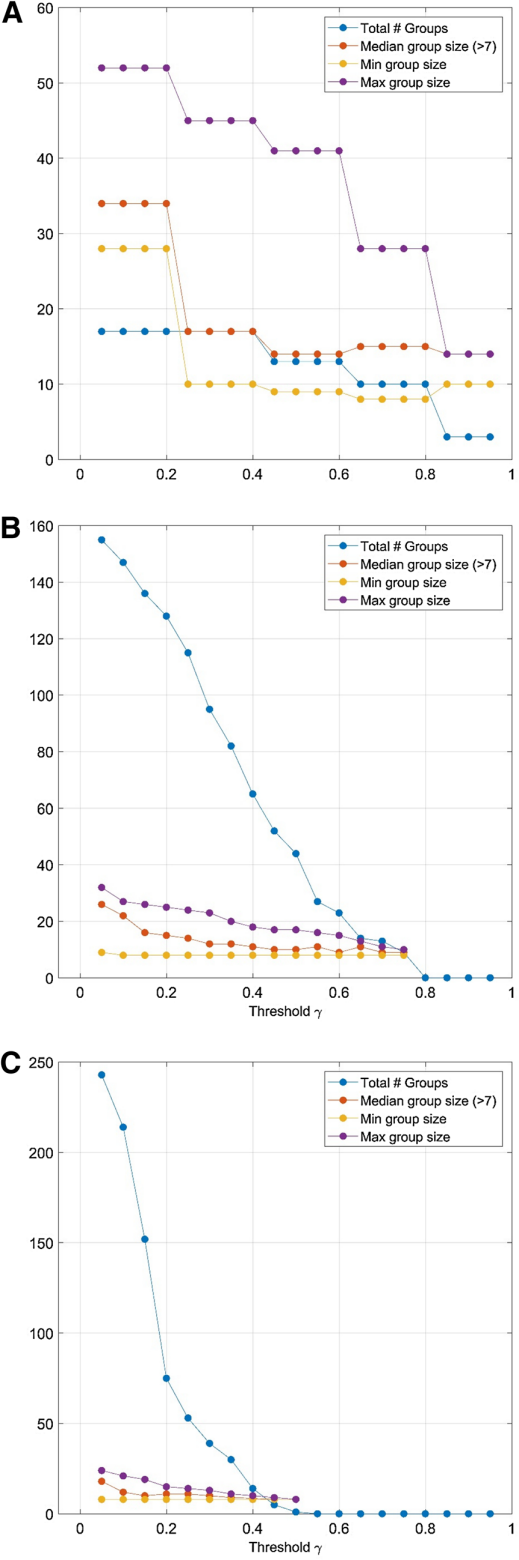
Number of groups $S_k$ and median number of variables per group as a function of $\gamma$ for the correlation maps (see Eq. 4 and accompanying text for more details) of: **a** factor 1, light condition; **b** factor 2, iime; **c** factor interaction (light condition $\times$ time)

The second step in GASCA is the selection of appropriate $\gamma$ values to control the sparsity of the solution. Since there are three maps, three values need to be chosen. Values can be selected by visually inspecting the map or by exploring the number of groups and their size as a function of $\gamma$: this is shown in Fig. 5. For the correlation map for factor 1 (light condition, panel a in Fig. 5) the median size of the $S_k$ groups is roughly constant for $\gamma> 0.4$; however the number of variables in the groups decreases sharply when increasing $\gamma$, as expected. We choose $\gamma _{\alpha }=0.85$ which gives a good compromise between the number of groups and their size, with not too many groups of moderate size. We use the subscript $\alpha$ to emphasize that this value of $\gamma$ is specific for the first factor, indicated with $\alpha$ in the models given by Eq. (7)

For factor 2 (Time, panel b in Fig. 5) the number of groups sharply decreases with $\gamma$ ( which indicates lower correlation among the variables) while the median, maximum and minim size remains approximately constant. We set $\gamma _{\beta }=0.7$ not to have too many groups. Both $\gamma _{\alpha }$ and $\gamma _{\beta }$ values are also in line with what can be inferred by visually inspecting the correlation plots from Fig. 4, like first suggested in the original publication of GPCA (Camacho et al. 2017)

For the interaction (light condition $\times$ Time, panel c in Fig. 5) the total number of groups decrease with $\gamma$ while the median, maximum and minimum size remains approximately constant. From the visual inspection of the correlation map in Fig. 4 panel c, it can be seen that there are very few groups of correlated variables, so we set $\gamma _{\alpha \beta } = 0.45$.

In general $\gamma$ should be set in such a way not to have too many groups containing only one or two variables. Because sparsity is an inherent property of the data also $\gamma$ is data specific and there is not a general rule to define the appropriate values which need to be specified with respect with the data at hand. However, since $\gamma$ is a threshold on the correlation magnitude, its value can be seen in context with what observed in metabolomics studies: Camacho (Camacho et al. 2005) suggested to divide correlations values into three levels: low ($|\rho | \le 0.6)$, medium $(0.6< |\rho | < 0.8)$, and high $(|| \ge 0.8)$ based on metabolic modeling considerations. In general, metabolomics data are abundant in low correlations as a result of the systemic nature of metabolic control (Camacho et al. 2005) and thus the low values observed for correlation map of the interaction terms are not unexpected.

Once the $\gamma$ values have been specified the GASCA model can be finally obtained: the loadings of the model (see Eqs. 7 and 11) are given in Table 1. Comparing the loadings of ASCA (Fig. 3) and GASCA models it is evident the gain in simplicity and interpretability of the solutions.

**Table 1:** Loadings for the two first components of GASCA model for the light condition and time effect matrices and their interaction

#	Metabolites	Factors and interactions					
		Light		Time		Light $\times$ Time	
		PC1	PC2	PC1	PC2	PC1	PC2
1	4-Hydroxy-benzoic acid				0.360		
2	4-Hydroxycinnamic acid				0.365		
3	Alanine						0.648
4	Arabinose						
5	Arabitol	0.186					
6	Ascorbic acid		− 0.061				
7	Asparagine	0.128		− 0.156			
8	Aspartate		− 0.549		− 0.195		
9	Benzoic acid		− 0.155	0.117			
10	Beta-alanine			− 0.491		− 0.268	− 0.071
11	Citramalate		− 0.256				
12	Citric acid						
13	Citrulline/arginine			− 0.084			
14	Dehydroascorbic acid		0.293				
15	Dehydroascorbic acid dimer						
16	Docosanoic acid				0.258		
17	Erythritol	0.114					
18	Ethanolamine	0.184			− 0.188		
19	Fructose		0.262				0.445
20	Fucose		− 0.124				
21	Fumaric acid	0.084					
22	Gaba	0.162				− 0.320	− 0.074
23	Galactinol				− 0.109		
24	Galactose						
25	Gluconic acid		− 0.228				
26	Glucose		0.315				0.580
27	Glutamate		− 0.164				
28	Glutamine		0.445				
29	Glycerol			0.202			
30	Glycolic acid	− 0.475					
31	Hexacosanoic acid		0.051				
32	Hydroxyproline						
33	Indole-3-acetonitrile						
34	Isoleucine			− 0.344		− 0.334	
35	Itaconic acid				0.336		
36	Lactic acid						
37	Leucine			− 0.371		− 0.369	
38	Lysine	0.214		− 0.352		− 0.449	− 0.005
39	Maleic acid						
40	Malic acid						
41	Maltose	0.061			− 0.355		
42	Mannitol						
43	Methionine	− 0.340				0.308	0.092
44	Myo-inositol						
45	Nicotinic acid						
46	O-acetyl-serine				0.254		
47	Octacosanoic acid						
48	Octadecanoic acid						
49	Ornithine						
50	Palmitic acid						
51	Phenylalanine	− 0.505			− 0.178		
52	Proline						
53	Putrescine				− 0.284		
54	Pyruvic acid						
55	Serine						
56	Shikimate	− 0.236				0.335	0.163
57	Similar to adenine						
58	Sinapic acid						
59	Succinic acid					− 0.251	
60	Sucrose	− 0.328		0.167			
61	Tetracosanoic acid				0.320		
62	Threonic acid		− 0.149				
63	Threonine						
64	Trehalose						
65	Tyrosine			− 0.405		− 0.325	
66	Uracil	0.244					
67	Valine		0.185	− 0.318			

Null loadings are omitted

**Fig. 6:**
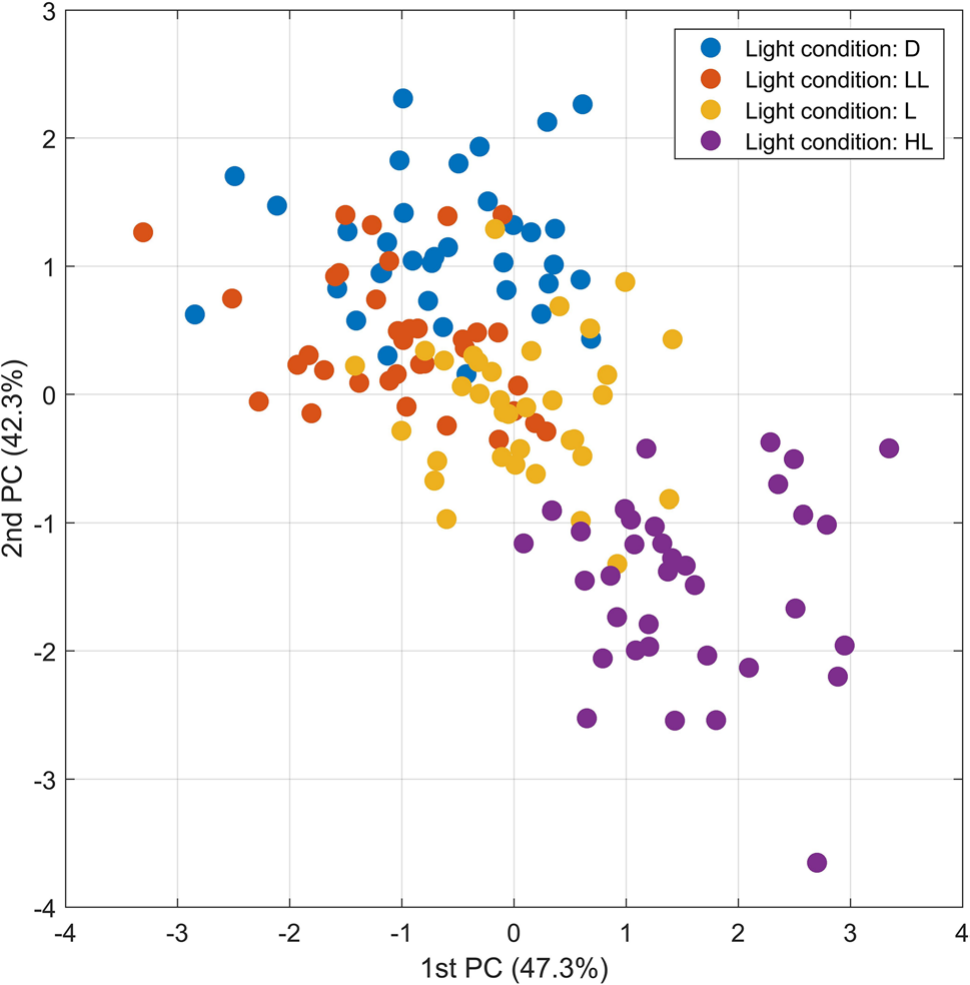
Score plot for the GASCA model for the first factor (light condition) of the plant data

The panel of measured metabolites analyzed here covers only a tiny fraction of the thousands of low molecular weight compounds produced by plants. However, several interesting observations can be made by analyzing the loadings (see Table 1), which describe the relative contribution of each variable to explain the variation observed in the data, associated to the different metabolites in the GASCA model. The score plot for the light condition factor is given in Fig. 6. The plot is slightly dissimilar from the one obtained for the standard ASCA model (see Fig. 3): however, separation among the different factor levels is evident, it should be remember that only subsets of variables are used in GASCA, hence differences will be observed among ASCA and GASCA score plots while interpretability is increased.

The loadings of the first component for the GASCA model for the light condition factor indicate substantial contribution of phenylalanine and shikimate whose pathways are indeed strongly interlinked (Tohge et al. 2013) and found to be affected by light (Caldana et al. 2011). Glycolic acid, a product of photosynthesis (Jensen and Bassham 1966), has also a high loading and can be an indicator of varying photosynthetic activity depending on the light levels.

The second components contains contributions from sugars (glucose, fructose, fucose), which is not surprising since plants transform carbon dioxide into sugars which are then used as energy source. Notably glutamine has here the larger loading, and this may indicate reduced glutamine synthetase activity, which plays a role in the control of photosynthetic responses to high light (Brestic et al. 2014).

Arginine is metabolically connected to glutamate: glutamate is used to synthesize ornithine from which arginine synthesis follows with citrulline as intermediate (Winter et al. 2015). Interestingly, ornithine does not contribute to the model indicating that likely the variance in the enzymes that control the reaction affects both metabolites in equal amounts and different directions resulting in very low correlation among these metabolites. Overall, the arginine biosynthesis in plant is poorly understood (Winter et al. 2015) and light modulating effects have been suggested (Frémont et al. 2013).

Concerning the factor Time (minutes of growth under a given light condition), the first component is dominated by benzoic acid whose synthesis pathway in plants has yet to be fully characterized (Moerkercke et al. 2009). This component contains also the branched amino acid leucine, isoleucine and valine whose biosynthesis in plants follows the reaction pathways established in microorganisms (Binder 2010) and play a pivotal role in plant development (Singh 1998)

Analysis of the loadings for the first two components for the interaction effect light condition $\times$ Time shows that succinate and leucine, isoleucine and methionine have high loading contribution and this indicates a link between amino acids and the tricarboxylic acid (TCA) cycle via succinate which is both light and time dependent. This suggests that amino acids produced by an increased protein biosynthesis may be used to fuel central metabolism (Caldana et al. 2011). Interestingly, also the 4-aminobutyric acid (GABA) has also high loading which suggests a possible role of this compound in fueling the TCA cycle. Using a differential network approach, Caldana and coworkers also highlighted GABA and suggested, building on a previous study (Taylor et al. 2004), that branched amino acids may promote their own degradation to acetyl-CoA, propionyl-CoA and acetoacetate that can subsequently enter the TCA cycle.

It is interesting to note that lysine is the only metabolite that appears in the models for all three effect matrices, thus providing a link between the plant response to light condition, plant development and their interaction. Indeed, it has been shown that lysine metabolism is strongly associated with the functioning of the tricarboxylic acid cycle while being largely disconnected from other metabolic networks (Angelovici et al. 2009): lysine catabolism into the TCA cycle seems to be fundamental for seed and plant development (Galili et al. 2014).

### GASCA analysis of the human data set

**Fig. 7:**
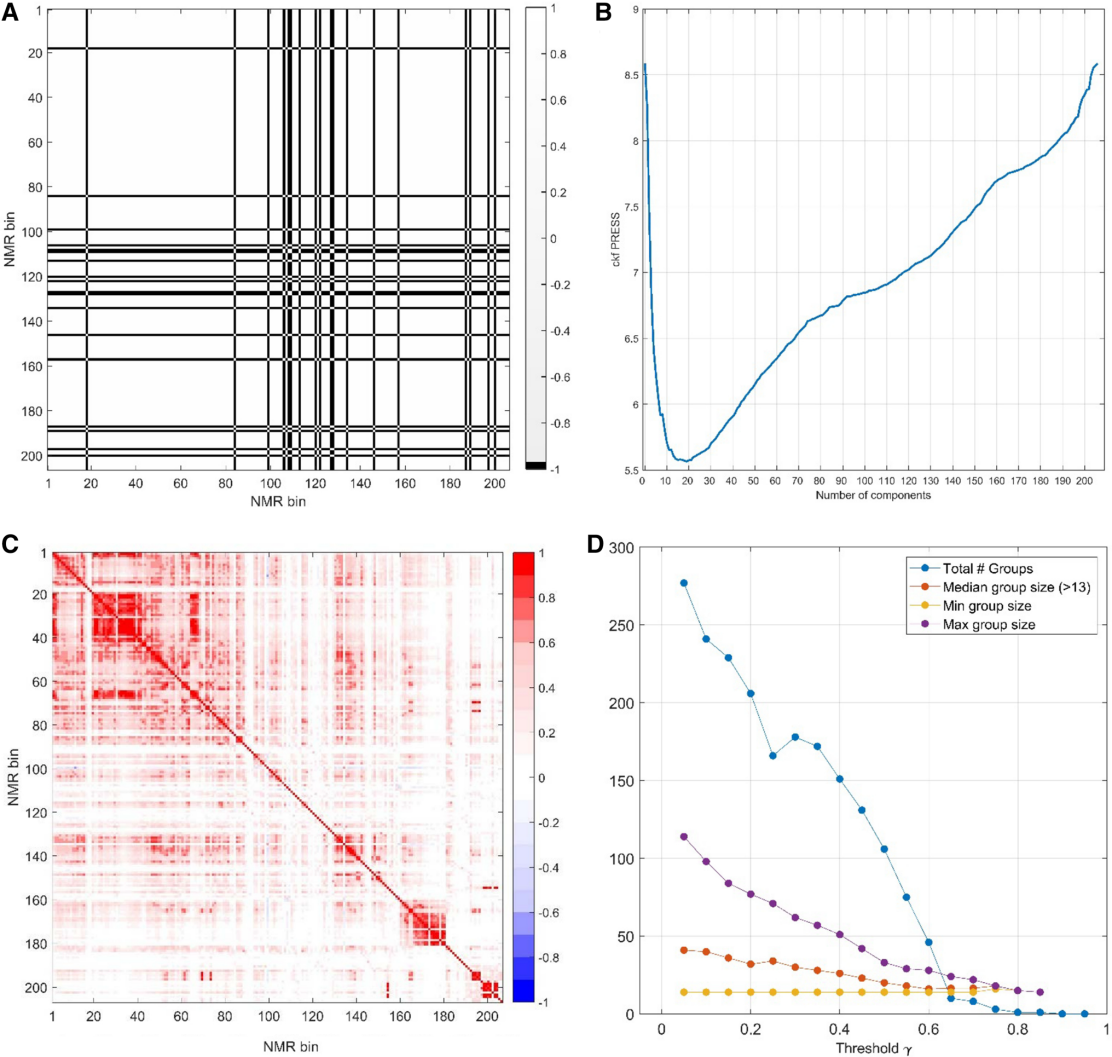
**a** Correlation matrix obtained from the effect matrix for the human data. **b** Cross-validation plot for the residual matrix (see Eq. in the main text.) **c** MEDA map fitted with 20 components. **d** Number of groups $S_k$ and median number of variables per group as a function of $\gamma$

We present here the analysis of the second experimental data set using GASCA. The experimental design follow a 1-factor ANOVA model with just two levels (subject 1 and 2). In this case is not possible to define a meaningful correlation matrix starting from the effect matrix as noted in the Sect. 2, which is shown in Fig. 7 panel a. For this reason we built the variable association map $\mathbf {M}$ starting from the matrix

9 $$\begin{aligned} \mathbf {E}_\alpha = \mathbf {X}- \mathbf {1}\mathbf {m}^{\mathrm {T}}, \end{aligned}$$

which the analogue for one-way design of Eq. (8), using the MEDA approach. To determine the optimal number of components to fit the MEDA map we use cross-validation, but other approaches are possible. Figure 7 panel b shows the cross-validation plot, from which we infer 20 to be the optimal dimensionality. This is used to obtain the MEDA map (see Eq. 3) shown in Fig. 7 panel c. As typical for NMR data sets, there is a high degree of correlations: the number of groups sharply decreases with the threshold $\gamma$ and we opt here for a rather sparse model by selecting $\gamma =0.6$, as shown in Fig. 7 panel d.

**Fig. 8:**
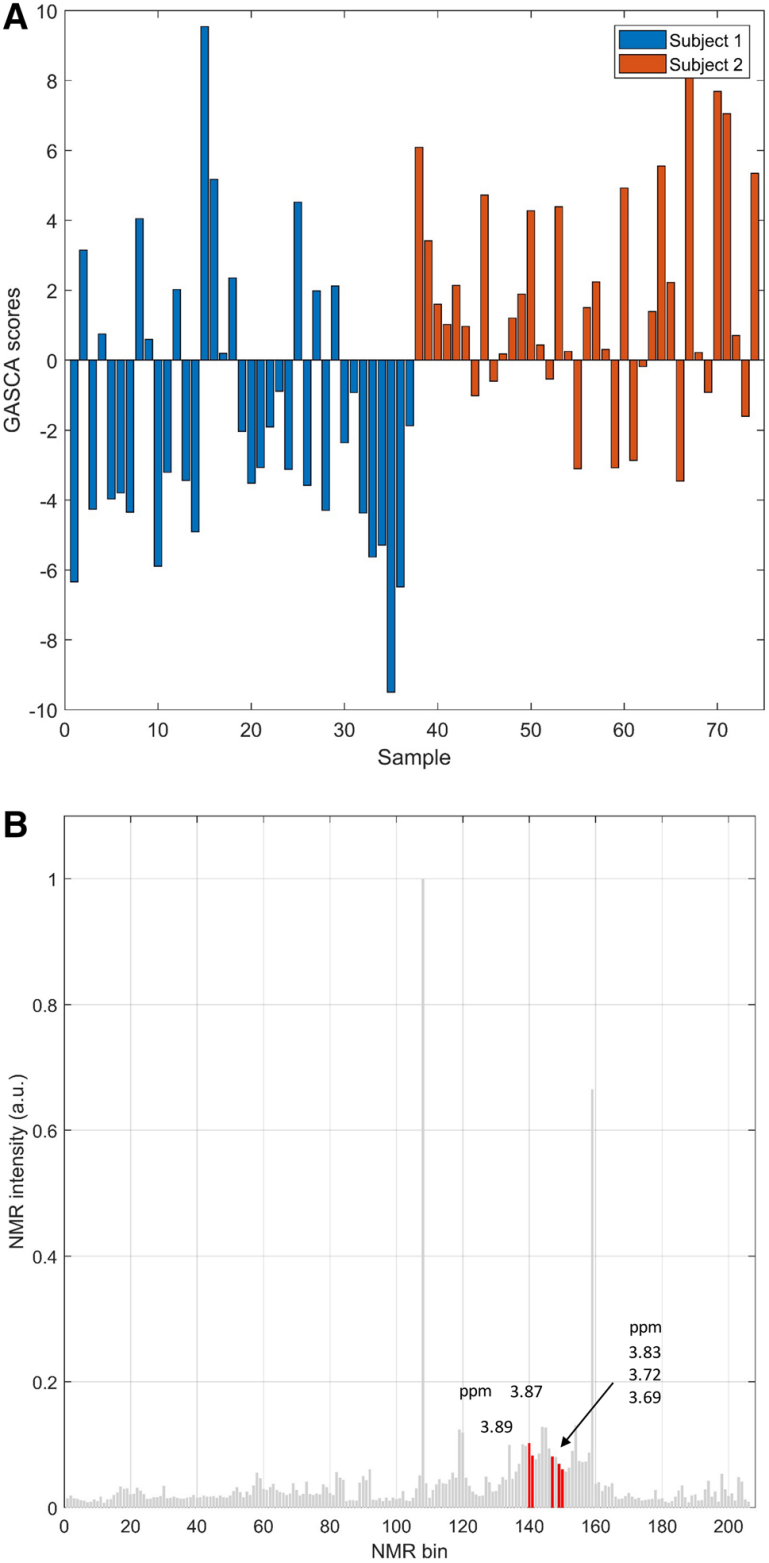
**a** Scores for the GASCA model for the human data. **b** Average NMR spectrum: the bins/ppm corresponding to the non-zero loadings for the GASCA component are highlighted in red

Since the design has only two levels, there is only one component in the GASCA model for this data. The monodimensional scores are shown in Fig. 8 panel a where it is evident the separation among the scores corresponding to the NMR spectra belonging to the Subject 1 and 2. The loadings for this component are shown in Fig. 8 panel b: a few ppm are selected (3.69, 3.71, 3.83, 3.87, and 3.89) which correspond to signal from dimethylglycine, citrate, trimethylamine and $\alpha$-ketoglutarate. Citrate and $\alpha$-ketoglutarate are intermediate of the TCA cycle. Dimethylglycine and trimethylamine are two metabolites associated, among others, with the activity of gut microflora, confirming the role of gut microflora activity to the shaping of the individual urinary metabolic phenotype (Bernini et al. 2009; Saccenti et al. 2016).

## Conclusions

Designed *omics* experiments are becoming increasingly complex with many factors considered simultaneously and having high dimensional multivariate responses. We have proposed here Group-wise ANOVA simultaneous component analysis (GASCA), an extension of the well established ANOVA-simultaneous component analysis (ASCA), which implements the idea of group-wise sparsity to arrive to solutions which are easier to interpret. The use of GASCA is advisable when data is sparse in a group-wise fashion, that is when there are groups of correlated/associated variables: this can be easily checked by visually inspecting the association maps built from the data. In this case GASCA models are easier to interpret than the ASCA counterpart.

The characteristics of the method are shown through the analysis of a real-life metabolomics experiments concerning the growth of *Arabidopsis thaliana* under different light conditions and phenotyping of healthy subjects. Results are compared with those of classical PCA and ASCA. It is shown that the GASCA models, containing only selected subsets of the original variables, are easier to interpret and describes relevant biological processes. We showed how the selection of closely related variable points to biologically relevant effects that are otherwise lost when all variables are considered. Finally, GASCA is applicable to any kind of *omics* data obtained through designed experiments such as, (but not limited to) gene expression and proteomic data.

## Appendix

Once the set of groups of variables $S_1, S_2, \ldots S_k, \ldots S_K$ have been identified, for instance with MEDA and GIA, the algorithmic procedure to obtain a GPCA model from the data matrix $\mathbf {X}$ is as follows:

Step 1 Initialize
  $$\begin{aligned} \mathbf {C}= & {} \mathbf {X}^\mathrm {T}\mathbf {X}\\ \mathbf B= & {} \mathbf {I}\end{aligned}$$
  where $\mathbf {C}$ has dimension $J\times J$ and **I** is a $J\times J$ identity matrix.
Step 2 For the *a*-th component with $a=1,2,\dots ,A$
  Step 2.1 For the *k*-th group $S_k$ of correlated variables $S_k$, with $k=1,2,\dots ,K$
  Step 2.1.1 Build the matrix $\mathbf {C}^k$ from $\mathbf {C}$ by setting to zero the variables not belonging to $S_k$ group.
    10 $$\begin{aligned} c^k_{lm} = 0, \ \forall l \not \in S_k \ or \ \forall m \not \in S_k \end{aligned}$$
    where $c^k_{lm}$ is the *l*-th, *m*-th element of $\mathbf {C}^k$
  Step 2.1.2 Perform eigendecomposition of $\mathbf {C}^k$ and select the first eigenvector.
    $$\begin{aligned} \mathbf {C}^k = \mathbf p ^k (\sigma ^k)^2 (\mathbf p ^k)^{\mathrm {T}} + \mathbf {E}^k \end{aligned}$$
    Step 2.2: Choose the loadings and scores of component *a* from the group capturing the most variance.
    11 $$\begin{aligned} \mathbf p _a= & {} \mathrm {argmin}_\mathbf{p ^k} \Vert \mathbf E ^k \Vert _F\end{aligned}$$
    12 $$\begin{aligned} \mathbf t _a= & {} \mathbf X {} \mathbf p _a \end{aligned}$$
    Step 2.3: Perform the deflation according to (Mackey, 2008).
    $$\begin{aligned} \mathbf q= & {} \mathbf {B}\mathbf p _a\\ \mathbf C= & {} (\mathbf {I}-\mathbf q {} \mathbf q ^{\mathrm {T}})\mathbf C (\mathbf I -\mathbf q {} \mathbf q ^{\mathrm {T}})\\ \mathbf X= & {} \mathbf X (\mathbf I -\mathbf q {} \mathbf q ^{\mathrm {T}})\\ \mathbf B= & {} \mathbf B (\mathbf I -\mathbf q {} \mathbf q ^{\mathrm {T}}) \end{aligned}$$
    Per each component, the GPCA algorithm computes *K* potential loading vectors, each of them with non-zero elements only for the set of variables corresponding to group $S_k$. To do that, it discards all the elements of the covariance matrix that do not correspond to variables in $S_k$ and performs a rank-1 eigen-decomposition on the resulting covariance. Comparing the resulting *K* eigenvectors, it selects the one with the highest variance, discarding the rest. Using this loading vector, the complete matrix **C** and the data matrix is deflated following (Mackey, 2008) to continue with successive components.
